# Endurance Exercise Attenuates Established Progressive Experimental Autoimmune Encephalomyelitis and Is Associated with an Amelioration of Innate Immune Responses in NOD Mice

**DOI:** 10.3390/ijms242115798

**Published:** 2023-10-31

**Authors:** Daniel Schiffmann, Victoria Lampkemeyer, Maren Lindner, Ann-Katrin Fleck, Kathrin Koch, Melanie Eschborn, Marie Liebmann, Jan-Kolja Strecker, Jens Minnerup, Heinz Wiendl, Luisa Klotz

**Affiliations:** Department of Neurology with Institute of Translational Neurology, University Hospital Muenster, 48149 Muenster, Germany; d_schi23@uni-muenster.de (D.S.);

**Keywords:** experimental autoimmune encephalomyelitis, multiple sclerosis, autoimmunity, central nervous system, exercise, lifestyle intervention

## Abstract

Multiple sclerosis (MS) is a chronic inflammatory autoimmune disease causing axonal degeneration and demyelination. Exercise in mice with active monophasic experimental autoimmune encephalomyelitis (EAE) attenuates disease severity associated with diverse impacts on T cell-mediated immunity. However, studies have so far focused on preventive approaches. In this study, we investigated the impact of endurance exercise on established EAE disease in a model of secondary progressive MS. When the exercise program on motorized running wheels was started at disease manifestation, the disease course was significantly ameliorated. This was associated with a significant decrease in B cell, dendritic cell, and neutrophil cell counts in the central nervous system (CNS). Furthermore, we observed an increased expression of major histocompatibility complex class II (MHC-II) as well as alterations in costimulatory molecule expression in CNS B cells and dendritic cells. In contrast, T cell responses were not altered in the CNS or periphery. Thus, exercise training is capable of attenuating the disease course even in established secondary progressive EAE, potentially via modulation of the innate immune compartment. Further studies are warranted to corroborate our findings and assess the potential of this lifestyle intervention as a complementary therapeutic strategy in secondary progressive MS patients.

## 1. Introduction

Multiple sclerosis (MS) is a chronic inflammatory autoimmune disease of the central nervous system (CNS), causing demyelination and axonal degeneration. Globally, more than 2 million people suffer from MS, with incidence and prevalence further increasing. While efficacious treatments for relapsing-remitting MS have been established, therapeutic options for secondary-progressive MS (SPMS) are still scarce [1,2,3,4].

The exact cause of multiple sclerosis remains elusive; however, the interplay of genetic susceptibility and a wide array of environmental factors influences the onset and clinical presentation of MS [3]. As such, low physical activity has been suggested as a risk factor for developing MS [5]. Physical activity in MS patients is lower compared to healthy individuals, yet patients who exercise reap diverse benefits, such as improvements in cardiorespiratory fitness, muscle strength, gait, balance, fatigue, and quality of life [6,7]. It is still debated whether exercise acts as a disease-modifying therapeutic intervention affecting the inflammatory pathophysiology or whether it primarily improves disease manifestations caused by previously decreased physical activity [8]. Therefore, while the positive impact of the potent anti-inflammatory effects of regular exercise on numerous diseases is known, the reason for the benefit of exercise in MS remains unclear. Additionally, an optimal exercise program remains undefined, given limited research in this area [9,10,11].

Exercise intervention studies in the MS animal model experimental autoimmune encephalomyelitis (EAE) have unraveled some disease-modifying properties of exercise. Souza et al. [12] reported delayed EAE onset as well as attenuated ensuing disease in EAE mice conducting either endurance or strength exercise (EE/SE), with EE being superior to SE. Decreased disease severity was associated with a decrease in oxidative stress as well as decreased proinflammatory cytokine expression in the central nervous system and the spleen. Positive effects, especially of endurance exercise, were further described in ensuing studies where (high-intensity) EE led to decreased proliferation of encephalitogenic T cells and a reduction in demyelination and CNS tissue damage, proinflammatory cytokine expression and CNS immune cell infiltration [13,14,15,16,17]. These exercise intervention studies have, however, primarily been conducted with C57BL/6 and SJL mice with their acute, monophasic/relapsing-remitting disease courses. Furthermore, the studies followed a preventative strategy with exercise beginning before disease induction. Some existing studies where the exercise intervention only commenced at disease induction showed similar positive effects in delaying disease onset/clinical severity and decreasing immune cell infiltration and demyelination, though [18,19].

Our study had the goal of further increasing the clinical relevance of EAE exercise studies. By delaying the start of the exercise intervention until established clinical disease, patient settings were mirrored more realistically. Furthermore, we used the non-obese diabetic (NOD)-EAE model, widely described as a model of secondary progressive MS, though some variability regarding disease phenotype has been noted [20,21]. Disease manifestation in these mice is influenced by the type and amount of CNS antigen or additive agents used for disease induction, the respective NOD mouse strain, as well as age at disease induction. This results in discrepancies regarding the timepoint of transition into progressive disease or even the existence of secondary progressive disease phases [20,21,22,23,24]. Nevertheless, this model is the most widely used model of secondary progressive MS that is currently available. Notably, in our laboratory, the disease course of NOD-EAE indeed reflects the disease course of SPMS. Therefore, we used this mouse model to study the impact of therapeutic exercise on the pathophysiology of disease progression in established CNS autoimmunity.

## 2. Results

**Figure 1:**
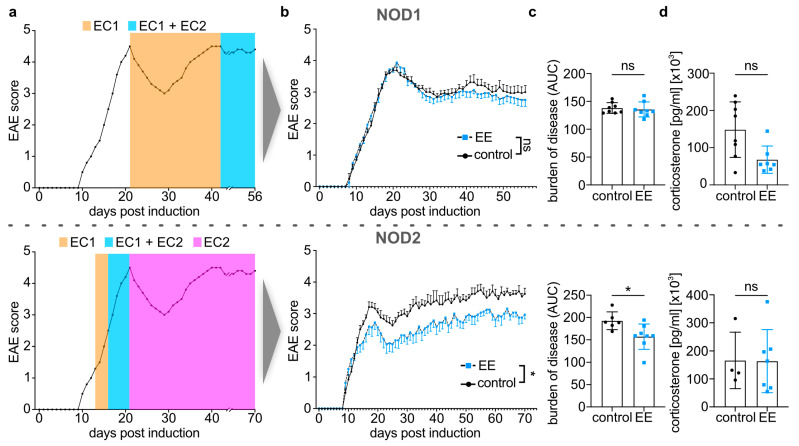
Controlled endurance exercise (EE) attenuated clinical disease in non-obese diabetic (NOD)-experimental autoimmune encephalomyelitis (EAE) mice. (**a**) Illustration of the NOD1 (**top**) and NOD2 (**bottom**) exercise programs over a schematic NOD-EAE disease course (see Section 4.4 and Figure S1 for a detailed outline of the exercise programs). (**b**) Disease courses of NOD-EAE mice for 56 days (NOD1; **top**) and 70 days (NOD2; **bottom**) post disease induction (dpi). Control mice (n = 8 (NOD1), n = 6 (NOD2)) were compared to mice conducting a controlled endurance exercise program (n = 8 for NOD1 and NOD2). (**c**) Comparison of the burden of disease (area under the curve) between NOD1 control (n = 8) and EE (n = 8) (**top**) and NOD2 control (n = 6) and EE (n = 8) (**bottom**) mice. (**d**) Comparison of serum corticosterone levels between NOD1 control (n = 8) and EE (n = 7) mice at 56 dpi (**top**) and NOD2 control (n = 4) and EE (n = 7) mice at 70 dpi (**bottom**). (**b**) EAE scores are shown as mean group scores ± SEM over time. In NOD2, pooled disease courses were significantly different, as determined by two-way ANOVA with Bonferroni correction. (**c**,**d**) Data are shown as mean ± SD. Each data point represents an individual mouse. Welch’s test or Mann–Whitney U tests were used for pairwise comparisons. * *p*-value ≤ 0.05. Abbreviations: exercise cycle 1 (EC1), exercise cycle 2 (EC2), area under the curve (AUC), non-significant (ns).

For exploring the diverse effects of endurance exercise (EE) interventions in the NOD-EAE model, we compared two different controlled endurance exercise programs (NOD1 and NOD2, respectively) as described in the methods section. Both exercise programs only began in established disease; the NOD1 program commenced at the peak of the relapse at 21 days post induction (dpi) and followed a less intense regimen. The NOD2 exercise program was more intense overall and was started earlier in the disease course at first signs of clinical disease at 13 dpi (Figure 1a) (see Section 4.4 and Figure S1 for a detailed outline of the exercise programs).

When comparing the disease course in both exercise programs, NOD2 mice clinically benefited from the exercise intervention with a significant (*p* < 0.05) disease amelioration compared to the control group. Such a positive effect was not observed in NOD1 mice (Figure 1b,c and Table S1). To exclude potential confounding effects caused by stress elicited by excessive exercise, we analyzed serum corticosterone levels as an established biomarker for stress assessment [25,26]. Though we did not find significant differences in serum corticosterone levels between EE and control groups in either NOD1 or NOD2 mice, NOD1 EE mice displayed a clear trend of decreased serum corticosterone levels compared to their respective controls. This trend was not observed in NOD2 EE mice (Figure 1d).

**Figure 2:**
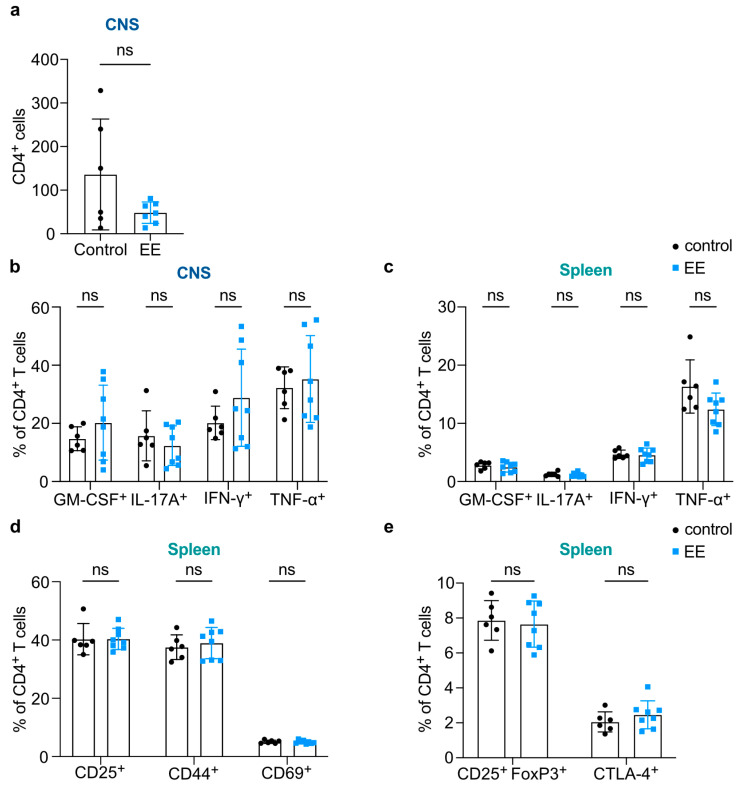
Endurance exercise (EE) had no influence on the CNS or peripheral CD4^+^ T cell response. (**a**) Comparison of CD4^+^ T cell counts in the CNS of NOD2 control (n = 6) and EE (n = 7) mice. (**b**) Proportions of CD4^+^ T cells producing proinflammatory cytokines in the CNS of NOD2 control (n = 6) and EE (n = 8) mice. (**c**) Proportions of CD4^+^ T cells producing proinflammatory cytokines in the spleen of NOD2 control (n = 6) and EE (n = 8) mice. (**d**) Proportions of CD4^+^ T cells expressing select activation markers in the spleen in NOD2 control (n = 6) and NOD2 EE (n = 8) mice. (**e**) Proportions of CD25^+^ FoxP3^+^ regulatory T cells and CD4^+^ T cells expressing CTLA-4 in the spleen of NOD2 control (n = 6) and EE (n = 8) mice. (**a**–**e**) Data are shown as mean ± SD. Each data point represents an individual mouse. Welch’s test or Mann–Whitney U tests were used for pairwise comparisons. Non-significant (ns).

Next, we examined whether the beneficial impact of endurance exercise on the EAE disease course in NOD2 EE mice was associated with changes in T cell responses in the periphery and/or the CNS. We observed a trend reduction in the absolute counts of CD4^+^ T cells in the CNS of the exercise group (Figure 2a). However, neither the percentage of cytokine (GM-CSF, IL-17A, IFN-γ, and TNF-α)-producing CD4^+^ T cells nor the *per-cell* levels of cytokine production as determined by mean fluorescence intensity (MFI) were found to be altered in the CNS of EE mice and control mice (Figure 2b and Figure S2). Likewise, in the spleen, we did not observe any differences between EE mice and controls except for a significantly (*p* ≤ 0.01) decreased *per-cell* TNF-α production (Figure 2c and Figure S2). In this mouse line, early activation markers CD25, CD44, and CD69 on splenic CD4^+^ T cells were not altered either (Figure 2d). Furthermore, we did not observe any changes in the frequencies of splenic CD4^+^ CD25^+^ FoxP3^+^ regulatory T cells (T_reg_ cells) as well as CD4^+^ CTLA-4^+^ T cells in these mice (Figure 2e). All in all, these results do not support any significant effect of endurance exercise on either effector or regulatory CD4^+^ T cell responses in the context of CNS autoimmunity.

**Figure 3:**
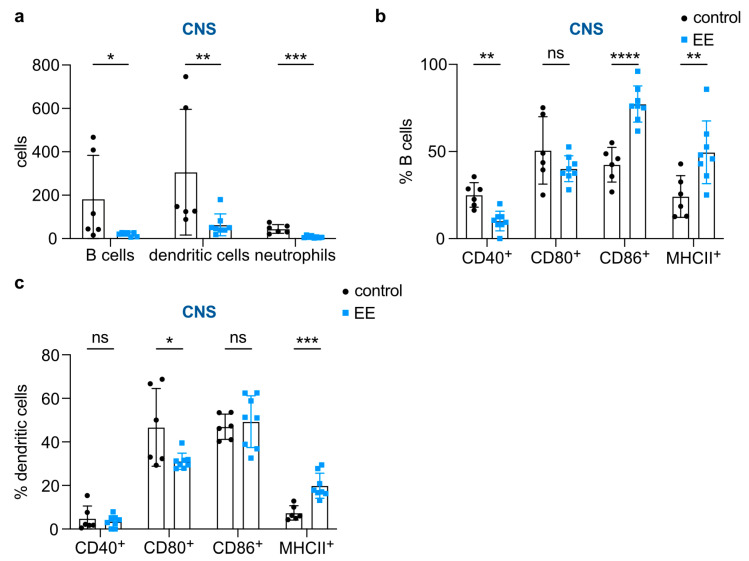
Endurance exercise (EE) reduced CNS abundance and influenced the costimulatory molecule expression of B cells and select innate immune cells. (**a**) CNS counts of B cells and select innate immune cells in NOD2 control (n = 6) and EE (n = 8) mice. (**b**) Proportions of B cells expressing costimulatory molecules in NOD2 control (n = 6) and EE (n = 8) mice in the CNS. (**c**) Proportions of dendritic cells expressing costimulatory molecules in NOD2 control (n = 6) and EE (n = 8) mice in the CNS. (**a**–**c**) Data are shown as mean ± SD. Each data point represents an individual mouse. Welch’s test or Mann–Whitney U tests were used for pairwise comparisons. * *p*-value ≤ 0.05; ** *p*-value ≤ 0.01; *** *p*-value ≤ 0.001; **** *p*-value ≤ 0.0001, non-significant (ns).

We next addressed whether EE may impact B cells and innate immune cell populations, including dendritic cells and neutrophils. Indeed, we observed a striking reduction in the absolute numbers of these cell populations in the CNS of EE mice compared to control mice (Figure 3a). We further investigated the functional properties of these cell populations by analyzing the expression levels of costimulatory molecules and major histocompatibility complex class II (MHC-II). Notably, for B cells, we observed a significant reduction in the expression of CD40, while CD86 and MHC-II were reciprocally increased (Figure 3b). In dendritic cells, we observed a significant decrease in CD80 and a reciprocal increase in MHC-II (Figure 3c). Together, these data suggest that endurance exercise impacts innate immune cell populations towards a more tolerogenic phenotype with strong antigen-presenting capacities and at least partly reduced costimulatory molecule expression.

## 3. Discussion

Previous studies have already described the beneficial effects of exercise in acute monophasic EAE, and these effects were, at least in part, linked to reduced proinflammatory T cell responses as well as increased peripheral immune cell tolerance [12,14,16]. With our study, we wanted to elucidate the potential effects of exercise on already established disease during the phase of disease progression using a mouse model of secondary progressive MS. Indeed, we could demonstrate that a more intense exercise program that was initiated at the timepoint of disease manifestation had a significant beneficial effect on the subsequent disease course in these mice. Notably, neither exercise program induced excessive stress, at least based on corticosterone levels in these mice. However, with their more exhausting exercise regimen, NOD2 EE mice did not benefit from the same stress-relieving trend of corticosterone reduction that we observed in NOD1 EE mice. Notably, we did not observe any alterations in effector and regulatory T cell profiles, both in the periphery and within the CNS. Instead, we observed profound effects of endurance exercise on B cells and dendritic cells within the CNS, both regarding their absolute counts and their activation status.

The marginal effect of our exercise intervention on T cell responses was surprising, considering that a positive effect of exercise on T cell proliferation and effector T cell responses in the CNS and periphery of EAE mice has been described [15,27,28]. Furthermore, several studies have demonstrated the influence of exercise programs on peripheral and/or central T_reg_ numbers [15,28,29]. In contrast, other studies did not observe an effect of endurance exercise on T_reg_ populations in EAE [12,13,27]. These discrepancies may be caused by differences in exercise protocol intensity and exercise modality, a different starting point of the exercise in the disease course, or differences in the animal model and the corresponding mouse strain, respectively. However, the fact that we observed robust disease amelioration despite unaltered effector and regulatory T cell responses suggests that the beneficial effects of exercise on disease progression in our animal model are not mediated by modulation of T cell responses. Current concepts of the pathophysiology of disease progression in MS focus on the increasing role of more compartmentalized inflammation within the CNS, primarily driven by innate immune cell populations [30,31]. From a clinical point of view, it should be noted that in SPMS patients, treatment strategies targeting T cell responses are of rather limited value, again supporting the notion that targeting T cell responses may not be an appropriate treatment approach for tackling disease progression [31].

A few studies have investigated the effects of exercise training on the innate immune system and B cells in EAE mice. As such, our findings of a profound reduction in B cells as well as innate immune cell populations are in line with published literature. Mice show a decrease in circulating B cells or neutrophils following several weeks of aerobic exercise, for example [32]. In C57BL/6 EAE mice, reduced spinal cord B cell infiltration in exercising groups has been described [33]. Interestingly, B cell depletion in EAE mice with established disease has been shown to alleviate disease severity. In contrast, B cell depletion prior to disease induction exacerbates ensuing EAE, thus supporting the significance of our finding of B cell reductions in progressive disease phases [34].

In contrast to the rather limited effects on T cells, we observed a profound effect on B cells as well as dendritic cells regarding cell numbers within the CNS and their activation profile. These findings suggest preferential effects of exercise training on the innate immune cell compartment, thereby positively influencing CNS autoimmunity. Although we observed diverse effects on costimulatory molecule expression on dendritic cells and B cells, our pattern indicates an overall reduction in the expression of the costimulatory molecules CD40 and CD80, while antigen-presenting function, as mirrored by MHC-II expression levels, was further enhanced. It has already been shown that CD80 plays an important role in both the induction and effector phases of EAE [35]. Furthermore, in the NOD-EAE model, the absence of either CD80 or CD86 results in an attenuated disease course [36]. Notably, in MS patients, the number of circulating B cells expressing CD80 is increased during relapse, and IFN-β 1b treatment reduces the number of circulating CD80-expressing B cells, suggesting a proinflammatory role of CD80 on B cells also in humans [37]. Increased expression of CD40 and its ligand CD40L has been described in MS patients, particularly during relapse, and has also been noted in EAE [38,39]. Here, a key role of CD40 in EAE pathogenesis has been highlighted through CD40 knockout studies where EAE induction was completely prevented [40]. Additionally, in non-human primate EAE studies, anti-CD40 monoclonal antibody administration, even after the onset of the disease, still had clinical benefit [41]. Based on these results, therapeutic targeting of the CD40-CD40L axis is currently being investigated as a potential treatment in clinical trials in MS [42].

The increased expression levels of MHC-II indicate preserved or even enhanced antigen presentation, i.e., signal 1 during T cell activation, while signaling via signal 2, i.e., co-stimulation, is concomitantly reduced, a constellation generally associated with immune tolerance [43,44,45].

Despite promising results on the effects of endurance exercise in NOD-EAE, our study had some limitations. These include a limited number of animals investigated, the score-inherent focus on motor symptoms while omitting other relevant functional systems, and the unphysiological timing of exercise during the daytime, thereby neglecting the physiological circadian rhythm of mice.

In conclusion, we here present evidence for a therapeutic endurance exercise program that attenuates clinical EAE severity in established disease using a mouse model of secondary progressive MS. Clinical disease attenuation was associated with quantitative and qualitative changes in the innate immune compartment, in particular dendritic cells and B cells, of exercising mice. Further studies are warranted to corroborate our findings and potentially establish this lifestyle intervention as a complementary immunomodulatory approach in patients with secondary progressive multiple sclerosis.

## 4. Materials and Methods

### 4.1. Mice

NOD/ShiLtJ mice were provided by The Jackson Laboratory (Stock No: 001976). Mice were housed in cages of four in a 12 h light/dark cycle with lights on at 6 am. They were kept under controlled environmental conditions (ambient temperature of 22 °C). Standard laboratory chow and tap water were allowed ad libitum. If mice lost more than 10% of their body weight relative to their initial body weight on day 0, they were given dietary supplements (Clear H_2_O, Westbrook, ME, USA). All animal experiments were performed according to the guidelines of the animal ethics committee and were approved by the local authorities of North Rhine-Westphalia, Germany (LANUV, AZ 81-02.04.2019.A478; approved on 15 April 2020).

### 4.2. EAE Induction

EAE was induced through active immunization, according to the published literature [46]. Mice were subcutaneously (s.c.) injected with 50 μg of myelin oligodendrocyte glycoprotein (MOG_35–55_) peptide (generated by Rudolf Volkmer, Charité Berlin, Germany) emulsified in Complete Freund’s Adjuvant (Difco by Becton, Dickinson and Company (BD), Sparks, MD, USA), including 500 μg of Mycobacterium tuberculosis H37Ra extract (Difco by BD, Sparks, MD, USA). On the day of immunization (day 0) and on day 2, mice were intraperitoneally (i.p.) injected with 200 ng Bordetella pertussis toxin (Sigma-Aldrich, St. Louis, MO, USA).

### 4.3. Disease Scoring

Mice were scored daily between 9 and 10 am according to the EAE score outlined in Table 1. Mice were excluded from the exercise intervention if the EAE score was 6 for >4 days or immediately if the EAE score was ≥7.

### 4.4. Exercise Intervention

Animals were randomly distributed into a control (n = 8) group with no exercise and an endurance exercise (EE) group (n = 8) that performed the controlled endurance exercise intervention. Exercise was performed in a motorized running wheel apparatus (Lafayette Instrument Company, Lafayette, IN, USA) for 73 min on five consecutive days per week. Exercise sessions consisted of 12 6-min interval exercise cycles and a 1-min cool-down period at low velocity (2 m/min). A slower exercise cycle 1 (EC1) with a maximum velocity of 6 m/min and a total distance of 377 m, and a faster exercise cycle 2 (EC2) with a maximum speed of 7 m/min and a total distance of 414 m were programmed in the Activity Wheel Monitor software (model 86065, Lafayette Instrument Company, Lafayette, IN, USA) of the running wheel apparatus (Figure S1a). Exercise was performed in the morning after clinical evaluation—a habituation period granted mice familiarization with the exercise protocol and motorized running wheels. Two different exercise programs with varying combinations of EC1 and EC2 and different start days were compared regarding their efficacy in influencing EAE disease in NOD mice. Exercise program 1 (NOD1) lasted for five weeks and started at the peak of the disease (21 days post induction (dpi)) with 12 EC1 cycles per session. At 42 dpi, daily exercise sessions were changed to a faster setup. Now, every session consisted of 36 min of EC2, followed by 37 min of EC1, covering a total distance of 396 m. NOD1 mice were euthanized at 56 dpi. Exercise program 2 (NOD2) started earlier in the disease course (disease manifestation defined as a score of every mouse ≥ 0.5), lasted longer, and was faster. At 13 dpi, sessions with only EC1 started. At 16 dpi, mice transitioned to the combined protocol of 36 min EC2 and 37 min EC1 until 21 dpi. From 21 dpi until euthanasia at 70 dpi, mice continued with only 12 cycles of EC2 (Figure S1b).

### 4.5. Isolation of Murine Leukocytes

Mice were sacrificed under deep anesthesia by intra-cardiac perfusion with PBS. Leukocytes were isolated from the CNS and spleen as previously described [47].

### 4.6. Quantitative Detection of Corticosterone

Corticosterone was quantified in serum acquired at respective days of euthanasia (56 dpi for NOD1 mice and 70 dpi for NOD2 mice) and was quantified using a corticosterone ELISA kit (Arbor Assays, Ann Arbor, MI, USA). The ELISA was performed according to the manufacturer’s instructions.

### 4.7. Flow Cytometry of Murine Leukocytes

All antibodies were obtained from BioLegend^®^ (San Diego, CA, USA) unless stated otherwise. Surface staining was conducted according to published literature [48]. The following antibodies were used: CD3 (clone: 17A2), CD4 (clone: GK1.5), CD11b (clone: M1/70), CD11c (clone: N418), CD25 (clone: PC61), CD40 (clone: 3/23, BD Biosciences, San Diego, CA, USA), CD44 (clone:IM7), CD45 (clone: 30-F11), CD45R (clone: RA3-6B2), CD69 (clone: H1.2F3), CD80 (clone: 16-10A1), CD86 (clone: GL-1), CD152 (clone: UC10-4B9), MHC-II (clone: M5/114.15.2), and mLy-6G (clone: 1A8). Intracellular staining was performed with the BD Cytofix/Cytoperm™ Fixation/Permeabilization Solution Kit (BD Biosciences, San Diego, CA, USA; 554714) according to the manufacturer’s instructions. The following antibodies were used: IL-17A (clone: TC11-18H10.1), IFN-γ (clone: XMG1.2), GM-CSF (clone: MP1-22E9), and TNF-α (clone: MP6-XT22). Intranuclear staining was done with eBioscience™ Fixation/Permeabilization Concentrate/Diluent (Invitrogen by Thermo Fisher Scientific, Carlsbad, CA, USA; 00-5123-43/ 00-5223-56) according to the manufacturer’s instructions. The following antibody was used: FOXP3 (clone: MF-14). Absolute cell counts were determined by CountBright™Absolute Counting Beads (Invitrogen by Thermo Fisher Scientific, Carlsbad, CA, USA). Data were acquired using a CytoFLEX S Flow Cytometer (Beckman Coulter, Krefeld, Germany). After appropriate compensation, common manual gating strategies were applied using Kaluza 1.5a software (Beckman Coulter, Krefeld, Germany). Absolute cell numbers, mean fluorescence intensity (MFI) (geometric mean), and percentages of respective subordinate populations were determined.

### 4.8. Statistical Analysis

Two-way ANOVA with Bonferroni correction was performed for the statistical evaluation of disease courses in line with published work [49]. Pairwise comparisons of continuous parametric data were performed using Welch’s t-test. Mann–Whitney U test was used for pairwise comparison of continuous non-parametric data. Statistical differences were considered significant when *p* < 0.05 (*). Statistical analyses were performed using GraphPad Prism 9 (GraphPad Software, Boston, MA, USA) software.

## Figures and Tables

**Table 1:** Description of the EAE score used for daily clinical disease assessment.

EAE Score	Comment
0	healthy
1	first clinical signs of ataxia, retained narrow gait, tip of tail dragging
2	slight waddle, complete tail dragging
3	wide gait, moderate ataxia, slight bilateral paralysis of the hindlimbs
4	uncoordinated gait with moderate bilateral paralysis of hindlimbs, lowered hindquarters
5	lowered and broad hindquarters, temporary dragging of one hind limb
6	permanent dragging of one hindlimb or both legs temporarily dragging
7	complete paralysis of the hindlimbs (paraplegia)
8	weakness of forelimbs, beginning tetraparesis
9	no movement, tetraplegia, altered breathing
10	moribund animal

## Data Availability

The datasets used/or analyzed during the current study are available from the corresponding author on reasonable request.

## Supplementary Materials

The following supporting information can be downloaded at: https://www.mdpi.com/article/10.3390/ijms242115798/s1.

## Abbreviations

CD	cluster of differentiation
CNS	central nervous system
CTLA-4	cytotoxic T lymphocyte antigen 4
dpi	days post induction
EAE	experimental autoimmune encephalomyelitis
EC1/2	exercise cycle 1/2
EE	endurance exercise
GM-CSF	granulocyte-macrophage colony-stimulating factor
IFN-β/γ	interferon β/γ
IL	interleukin
MFI	mean fluorescence intensity
MHC-II	major histocompatibility complex class II
MOG	myelin oligodendrocyte glycoprotein
MS	multiple sclerosis
NOD	non-obese diabetic
NOD1/2	exercise program 1/2
SE	strength exercise
SPMS	secondary progressive multiple sclerosis
TNF-α	tumor necrosis factor α

## References

[1] Ransohoff R.M., Hafler D.A., Lucchinetti C.F. (2015). Multiple sclerosis—A quiet revolution. Nat. Rev. Neurol..

[2] Browne P., Chandraratna D., Angood C., Tremlett H., Baker C., Taylor B.V., Thompson A.J. (2014). Atlas of Multiple Sclerosis 2013: A growing global problem with widespread inequity. Neurology.

[3] Dendrou C.A., Fugger L., Friese M.A. (2015). Immunopathology of multiple sclerosis. Nat. Rev. Immunol..

[4] Gholamzad M., Ebtekar M., Ardestani M.S., Azimi M., Mahmodi Z., Mousavi M.J., Aslani S. (2019). A comprehensive review on the treatment approaches of multiple sclerosis: Currently and in the future. Inflamm. Res..

[5] Cortese M., Riise T., Bjørnevik K., Myhr K.M., The Multiple Sclerosis Conscript Service Database Study Group (2018). Body size and physical exercise, and the risk of multiple sclerosis. Mult. Scler..

[6] Gold S.M., Schulz K.H., Hartmann S., Mladek M., Lang U.E., Hellweg R., Reer R., Braumann K.M., Heesen C. (2003). Basal serum levels and reactivity of nerve growth factor and brain-derived neurotrophic factor to standardized acute exercise in multiple sclerosis and controls. J. Neuroimmunol..

[7] Halabchi F., Alizadeh Z., Sahraian M.A., Abolhasani M. (2017). Exercise prescription for patients with multiple sclerosis; potential benefits and practical recommendations. BMC Neurol..

[8] Dalgas U., Stenager E. (2012). Exercise and disease progression in multiple sclerosis: Can exercise slow down the progression of multiple sclerosis?. Ther. Adv. Neurol. Disord..

[9] Flynn M.G., McFarlin B.K., Markofski M.M. (2007). The anti-inflammatory actions of exercise training. Am. J. Lifestyle Med..

[10] Coote S. (2014). Progressive resistance therapy is not the best way to rehabilitate deficits due to multiple sclerosis: Yes. Mult. Scler..

[11] Motl R.W., Sandroff B.M., Kwakkel G., Dalgas U., Feinstein A., Heesen C., Feys P., Thompson A.J. (2017). Exercise in patients with multiple sclerosis. Lancet Neurol..

[12] Souza P.S., Gonçalves E.D., Pedroso G.S., Farias H.R., Junqueira S.C., Marcon R., Tuon T., Cola M., Silveira P.C.L., Santos A.R. (2017). Physical exercise attenuates experimental autoimmune encephalomyelitis by inhibiting peripheral immune response and blood-brain barrier disruption. Mol. Neurobiol..

[13] Einstein O., Fainstein N., Touloumi O., Lagoudaki R., Hanya E., Grigoriadis N., Katz A., Ben-Hur T. (2018). Exercise training attenuates experimental autoimmune encephalomyelitis by peripheral immunomodulation rather than direct neuroprotection. Exp. Neurol..

[14] Fainstein N., Tyk R., Touloumi O., Lagoudaki R., Goldberg Y., Agranyoni O., Navon-Venezia S., Katz A., Grigoriadis N., Ben-Hur T. (2019). Exercise intensity-dependent immunomodulatory effects on encephalomyelitis. Ann. Clin. Transl. Neurol..

[15] Xie Y., Li Z., Wang Y., Xue X., Ma W., Zhang Y., Wang J. (2019). Effects of moderate- versus high- intensity swimming training on inflammatory and CD4+ T cell subset profiles in experimental autoimmune encephalomyelitis mice. J. Neuroimmunol..

[16] Hamdi L., Nabat H., Goldberg Y., Fainstein N., Segal S., Mediouni E., Asis Y., Touloumi O., Grigoriadis N., Katz A. (2022). Exercise training alters autoimmune cell invasion into the brain in autoimmune encephalomyelitis. Ann. Clin. Transl. Neurol..

[17] Sohrabi P., Parnow A., Jalili C. (2023). Treadmill aerobic training improve beam-walking test, up-regulate expression of main proteins of myelin and myelination in the hippocampus of cuprizone-fed mice. Neurosci. Lett..

[18] Le Page C., Bourdoulous S., Béraud E., Couraud P.O., Rieu M., Ferry A. (1996). Effect of physical exercise on adoptive experimental auto-immune encephalomyelitis in rats. Eur. J. Appl. Physiol. Occup. Physiol..

[19] Pryor W.M., Freeman K.G., Larson R.D., Edwards G.L., White L.J. (2015). Chronic exercise confers neuroprotection in experimental autoimmune encephalomyelitis. J. Neurosci. Res..

[20] Tanabe S., Saitoh S., Miyajima H., Itokazu T., Yamashita T. (2019). Microglia suppress the secondary progression of autoimmune encephalomyelitis. Glia.

[21] Colpitts S.L., Kasper E.J., Keever A., Liljenberg C., Kirby T., Magori K., Kasper L.H., Ochoa-Repáraz J. (2017). A bidirectional association between the gut microbiota and CNS disease in a biphasic murine model of multiple sclerosis. Gut Microbes.

[22] Dang P.T., Bui Q., D’Souza C.S., Orian J.M., La Flamme A.C., Orian J.M. (2015). Modelling MS: Chronic-relapsing EAE in the NOD/Lt mouse strain. Emerging and Evolving Topics in Multiple Sclerosis Pathogenesis and Treatments.

[23] Levy H., Assaf Y., Frenkel D. (2010). Characterization of brain lesions in a mouse model of progressive multiple sclerosis. Exp. Neurol..

[24] Baker D., Nutma E., O’Shea H., Cooke A., Orian J.M., Amor S. (2019). Autoimmune encephalomyelitis in NOD mice is not initially a progressive multiple sclerosis model. Ann. Clin. Transl. Neurol..

[25] da Rocha A.L., Pinto A.P., Kohama E.B., Pauli J.R., de Moura L.P., Cintra D.E., Ropelle E.R., da Silva A.S.R. (2019). The proinflammatory effects of chronic excessive exercise. Cytokine.

[26] Gong S., Miao Y.L., Jiao G.Z., Sun M.J., Li H., Lin J., Luo M.J., Tan J.H. (2015). Dynamics and correlation of serum cortisol and corticosterone under different physiological or stressful conditions in mice. PLoS ONE.

[27] Goldberg Y., Fainstein N., Zaychik Y., Hamdi L., Segal S., Nabat H., Touloumi O., Zoidou S., Grigoriadis N., Hoffman J.R. (2021). Continuous and interval training attenuate encephalomyelitis by separate immunomodulatory mechanisms. Ann. Clin. Transl. Neurol..

[28] Chen H., Shen L., Liu Y., Ma X., Long L., Ma X., Ma L., Chen Z., Lin X., Si L. (2021). Strength Exercise Confers Protection in Central Nervous System Autoimmunity by Altering the Gut Microbiota. Front. Immunol..

[29] Wang J., Song H., Tang X., Yang Y., Vieira V.J., Niu Y., Ma Y. (2012). Effect of exercise training intensity on murine T-regulatory cells and vaccination response. Scand. J. Med. Sci. Sports.

[30] Kuhlmann T., Moccia M., Coetzee T., Cohen J.A., Correale J., Graves J., Marrie R.A., Montalban X., Yong V.W., Thompson A.J. (2023). Multiple sclerosis progression: Time for a new mechanism-driven framework. Lancet Neurol..

[31] Klotz L., Antel J., Kuhlmann T. (2023). Inflammation in multiple sclerosis: Consequences for remyelination and disease progression. Nat. Rev. Neurol..

[32] Frodermann V., Rohde D., Courties G., Severe N., Schloss M.J., Amatullah H., McAlpine C.S., Cremer S., Hoyer F.F., Ji F. (2019). Exercise reduces inflammatory cell production and cardiovascular inflammation via instruction of hematopoietic progenitor cells. Nat. Med..

[33] Bernardes D., Oliveira-Lima O.C., Silva T.V., Faraco C.C., Leite H.R., Juliano M.A., Santos D.M., Bethea J.R., Brambilla R., Orian J.M. (2013). Differential brain and spinal cord cytokine and BDNF levels in experimental autoimmune encephalomyelitis are modulated by prior and regular exercise. J. Neuroimmunol..

[34] Wang A., Rojas O., Lee D., Gommerman J.L. (2021). Regulation of neuroinflammation by B cells and plasma cells. Immunol. Rev..

[35] Chang T.T., Jabs C., Sobel R.A., Kuchroo V.K., Sharpe A.H. (1999). Studies in B7-deficient mice reveal a critical role for B7 costimulation in both induction and effector phases of experimental autoimmune encephalomyelitis. J. Exp. Med..

[36] Girvin A.M., Dal Canto M.C., Rhee L., Salomon B., Sharpe A., Bluestone J.A., Miller S.D. (2000). A critical role for B7/CD28 costimulation in experimental autoimmune encephalomyelitis: A comparative study using costimulatory molecule-deficient mice and monoclonal antibody blockade. J. Immunol..

[37] Genç K., Dona D.L., Reder A.T. (1997). Increased CD80(+) B cells in active multiple sclerosis and reversal by interferon beta-1b therapy. J. Clin. Investig..

[38] Gerritse K., Laman J.D., Noelle R.J., Aruffo A., Ledbetter J.A., Boersma W.J., Claassen E. (1996). CD40-CD40 ligand interactions in experimental allergic encephalomyelitis and multiple sclerosis. Proc. Natl. Acad. Sci. USA.

[39] Issazadeh S., Navikas V., Schaub M., Sayegh M., Khoury S. (1998). Kinetics of expression of costimulatory molecules and their ligands in murine relapsing experimental autoimmune encephalomyelitis in vivo. J. Immunol..

[40] Becher B., Durell B.G., Miga A.V., Hickey W.F., Noelle R.J. (2001). The clinical course of experimental autoimmune encephalomyelitis and inflammation is controlled by the expression of CD40 within the central nervous system. J. Exp. Med..

[41] Laman J.D., ‘t Hart B.A., Brok H., Meurs M., Schellekens M.M., Kasran A., Boon L., Bauer J., Boer M., Ceuppens J. (2002). Protection of marmoset monkeys against EAE by treatment with a murine antibody blocking CD40 (mu5D12). Eur. J. Immunol..

[42] Fadul C.E., Mao-Draayer Y., Ryan K.A., Noelle R.J., Wishart H.A., Channon J.Y., Kasper I.R., Oliver B., Mielcarz D.W., Kasper L.H. (2021). Safety and Immune Effects of Blocking CD40 Ligand in Multiple Sclerosis. Neurol. Neuroimmunol. Neuroinflamm..

[43] Kwidzinski E., Mutlu L.K., Kovac A.D., Bunse J., Goldmann J., Mahlo J., Aktas O., Zipp F., Kamradt T., Nitsch R. (2003). Self-tolerance in the immune privileged CNS: Lessons from the entorhinal cortex lesion model. Advances in Research on Neurodegeneration. Journal of Neural Transmission. Supplementa.

[44] Bechmann I., Peter S., Beyer M., Gimsa U., Nitsch R. (2001). Presence of B-7.2 (CD86) and lack of B7-1 (CD80) on myelin-phagocytosing MHC-II positive rat microglia are associated with nondestructive immunity in vivo. FASEB J..

[45] Krovi S.H., Kuchroo V.K. (2022). Activation pathways that drive CD4(+) T cells to break tolerance in autoimmune diseases. Immunol. Rev..

[46] Klotz L., Burgdorf S., Dani I., Saijo K., Flossdorf J., Hucke S., Alferink J., Nowak N., Beyer M., Mayer G. (2009). The nuclear receptor PPAR gamma selectively inhibits Th17 differentiation in a T cell-intrinsic fashion and suppresses CNS autoimmunity. J. Exp. Med..

[47] Hucke S., Floßdorf J., Grützke B., Dunay I.R., Frenzel K., Jungverdorben J., Linnartz B., Mack M., Peitz M., Brüstle O. (2012). Licensing of myeloid cells promotes central nervous system autoimmunity and is controlled by peroxisome proliferator-activated receptor γ. Brain.

[48] Klotz L., Kuzmanov I., Hucke S., Gross C.C., Posevitz V., Dreykluft A., Schulte-Mecklenbeck A., Janoschka C., Lindner M., Herold M. (2016). B7-H1 shapes T-cell-mediated brain endothelial cell dysfunction and regional encephalitogenicity in spontaneous CNS autoimmunity. Proc. Natl. Acad. Sci. USA.

[49] Fleck A.K., Hucke S., Teipel F., Eschborn M., Janoschka C., Liebmann M., Wami H., Korn L., Pickert G., Hartwig M. (2021). Dietary conjugated linoleic acid links reduced intestinal inflammation to amelioration of CNS autoimmunity. Brain.

