# Supplementary material for: Endurance Exercise Attenuates Established Progressive Experimental Autoimmune Encephalomyelitis and Is Associated with an Amelioration of Innate Immune Responses in NOD Mice
Source: Int J Mol Sci. 2023 Oct 31;24(21):15798. doi: 10.3390/ijms242115798 (PMC10648469; doi:10.3390/ijms242115798)
Supplement: Supplementary file 1 [file ijms-24-15798-s001.zip › Figure S1.pdf]

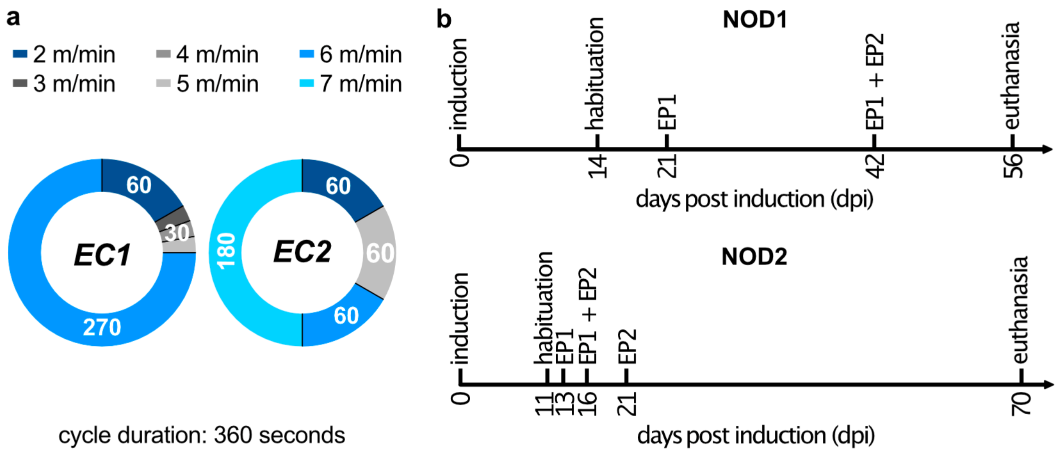

Figure S1. Schematic overview of the exercise program. (**a**) Exercise cycle 1 and 2 consisted of 12 6-minute cycles. Running wheel velocity within each cycle changed in intervals as indicated in the diagram (bold white numbers indicate time in seconds of each interval within one cycle). Each 72-minute exercise session was ensued by a 1-minute cool down period at 2 m/min. (**b**) Timeline of the different steps within the NOD1 (top) and NOD2 (bottom) exercise program in days post induction (dpi).
