# Supplementary material for: Endurance Exercise Attenuates Established Progressive Experimental Autoimmune Encephalomyelitis and Is Associated with an Amelioration of Innate Immune Responses in NOD Mice
Source: Int J Mol Sci. 2023 Oct 31;24(21):15798. doi: 10.3390/ijms242115798 (PMC10648469; doi:10.3390/ijms242115798)
Supplement: Supplementary file 1 [file ijms-24-15798-s001.zip › Figure S2.pdf]

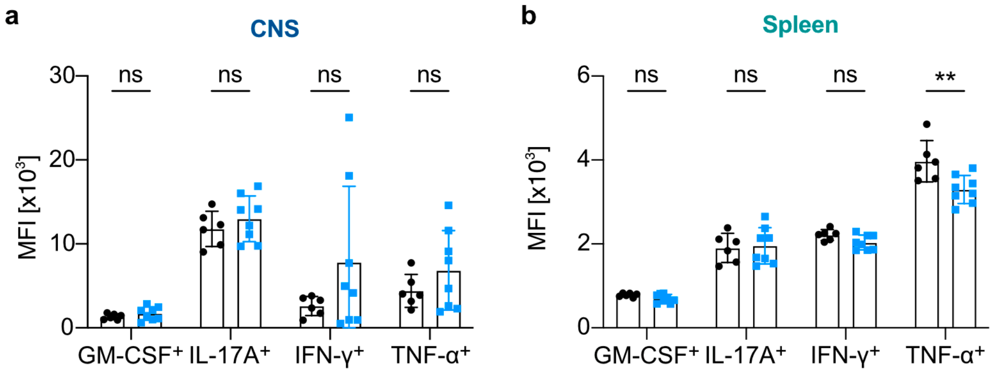

Figure S2. *Per-cell* cytokine production of CD4⁺ T cells (**a**) Mean fluorescence intensity (MFI) of CD4⁺ T cells producing proinflammatory cytokines in the CNS of NOD2 control (n = 6) and EE (n = 8) mice. (**b**) MFI of CD4⁺ T cells producing proinflammatory cytokines in the spleen of NOD2 control (n = 6) and EE (n = 8) mice. (**a,b**) Data are shown as mean ± SD. Each data point represents an individual mouse. Welch's test or Mann-Whitney U tests were used for pairwise comparisons. **p-value ≤ 0.01.
