# Supplementary material for: Endurance Exercise Attenuates Established Progressive Experimental Autoimmune Encephalomyelitis and Is Associated with an Amelioration of Innate Immune Responses in NOD Mice
Source: Int J Mol Sci. 2023 Oct 31;24(21):15798. doi: 10.3390/ijms242115798 (PMC10648469; doi:10.3390/ijms242115798)
Supplement: Supplementary file 1 [file ijms-24-15798-s001.zip › Table S1.pdf]

Table S1. Hallmarks of the clinical disease courses of both NOD1- and NOD2-EAE mice. Data represent the mean EAE score ± SD; *p-value ≤ 0.05; non-significant differences are not separately indicated.

	NOD1 control	NOD1 EE	NOD2 control	NOD2 EE
number of mice	8	8	8	8
number of eliminated mice	-	-	2	-
rate of successful disease induction	100 %	100 %	100 %	100 %
day of onset	9 ± 0.76	9 ± 0.54	9.2 ± 0.41	9.1 ± 0.35
peak score	3.7 ± 0.22	3.9 ± 0.32	3.2 ± 0.40	2.7 ± 0.62
remission score	2.8 ± 0.3	2.8 ± 0.43	2.6 ± 0.38	2 ± 0.69
end score	3 ± 0.58	2.8 ± 0.57	3.6 ± 0.44	3 ± 0.39 *
burden of disease (area under the curve)	138.1 ± 9.58	135.6 ± 13.56	192.7 ± 19.98	157.2 ± 28.17 *
