# Supplementary figures and images for: Endurance Exercise Attenuates Established Progressive Experimental Autoimmune Encephalomyelitis and Is Associated with an Amelioration of Innate Immune Responses in NOD Mice
Source: Int J Mol Sci. 2023 Oct 31;24(21):15798. doi: 10.3390/ijms242115798 (PMC10648469; doi:10.3390/ijms242115798)

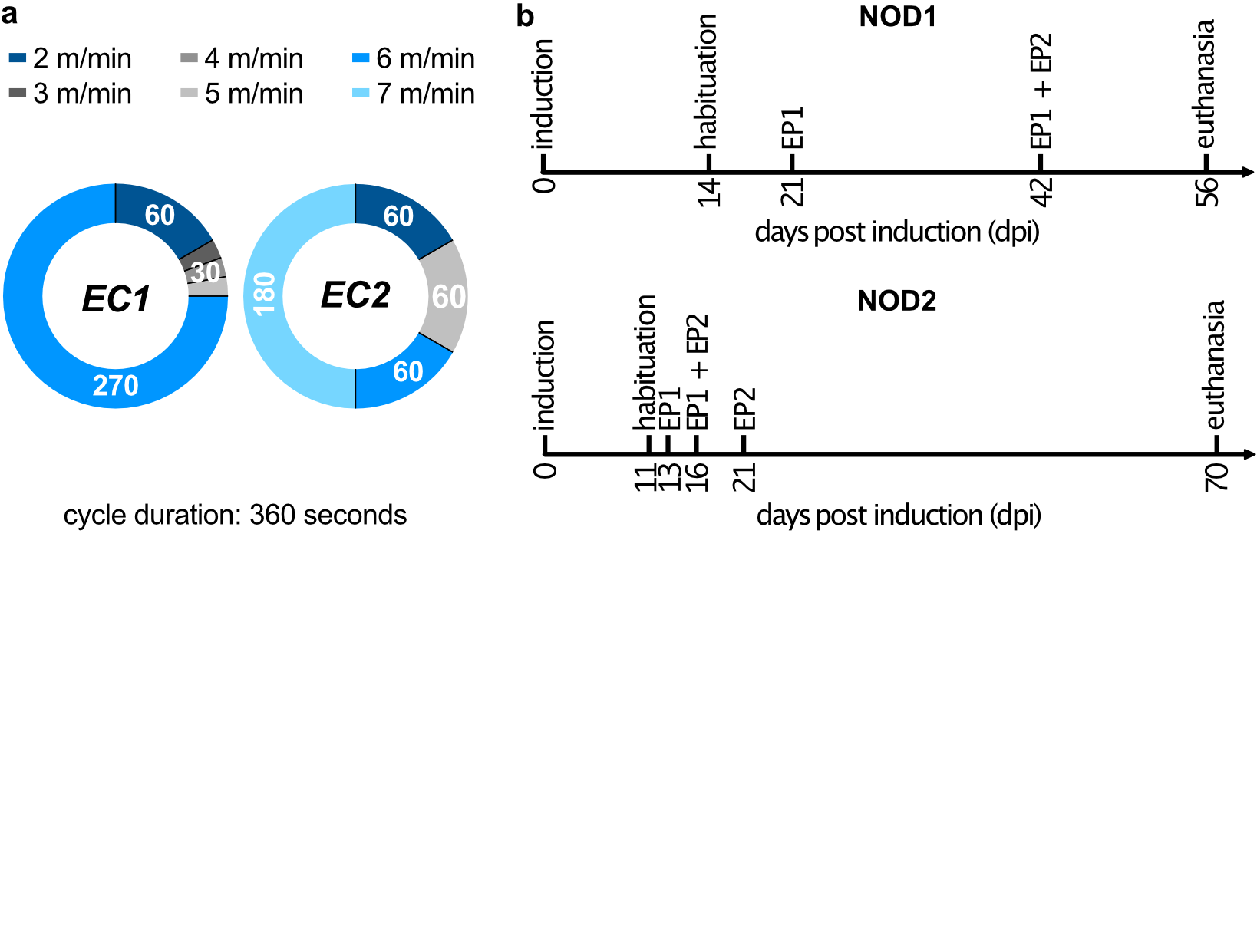

Supplement: Supplementary file 1 [file ijms-24-15798-s001.zip › Supplementary Figure S1 NOD EAE Paper.tiff]

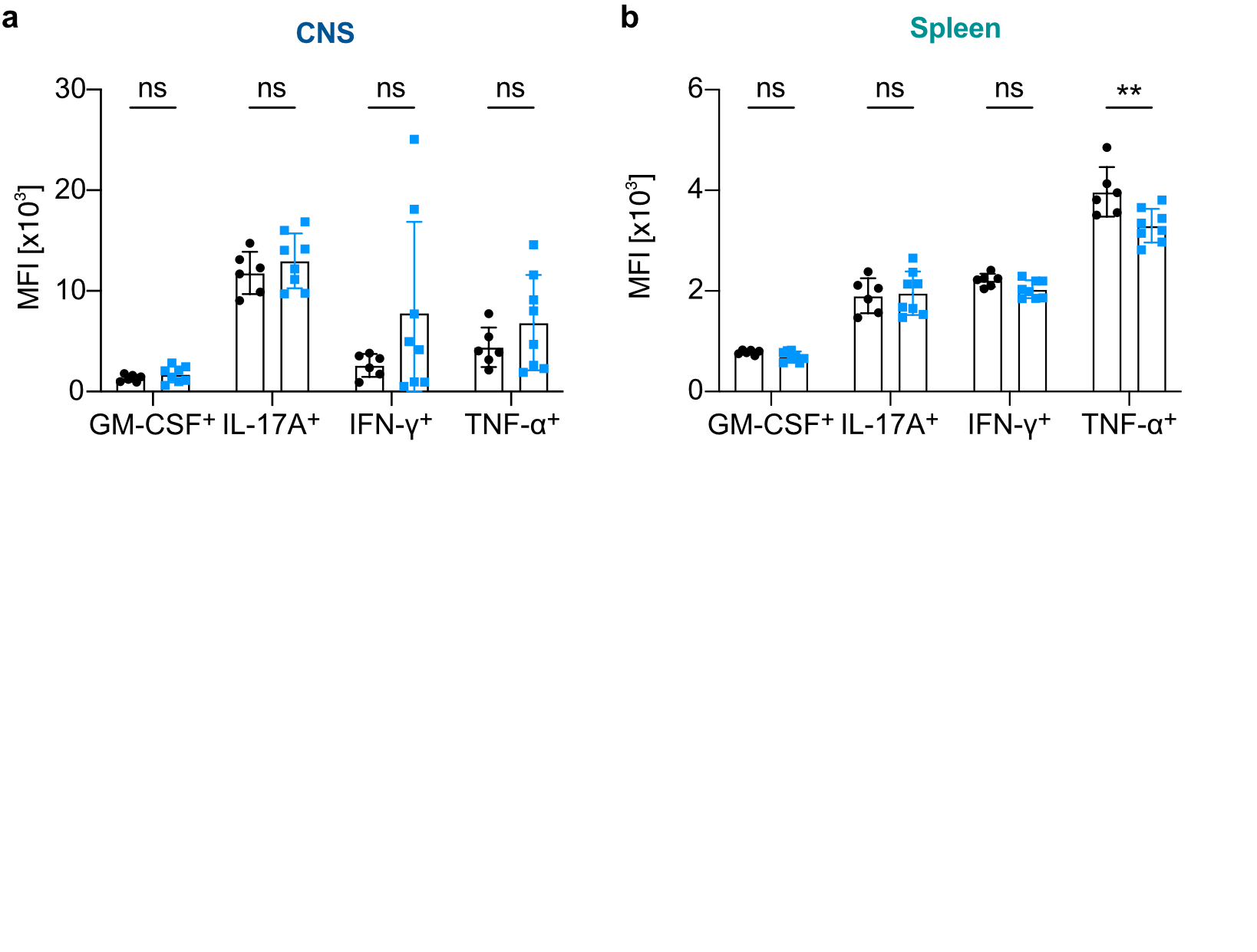

Supplement: Supplementary file 1 [file ijms-24-15798-s001.zip › Supplementary Figure S2 NOD EAE Paper.tiff]
